# Inside the Black Box: Organisational Buying Behaviour and Strategic Purchasing in Healthcare: A Response to Recent Commentary

**DOI:** 10.15171/ijhpm.2019.70

**Published:** 2019-09-07

**Authors:** Joe Sanderson, Chris Lonsdale, Russell Mannion

**Affiliations:** ^1^Birmingham Business School, University of Birmingham, Birmingham, UK.; ^2^Health Services Management Centre, University of Birmingham, Birmingham, UK.

## Introduction

We read the Hanson et al^1^ paper with great interest, utilising as it does our *IJHPM* paper, ‘What’s Needed to Develop Strategic Purchasing in Healthcare? Policy Lessons from a Realist Review.’^2^ We very much agree with their statement identifying a key gap in our paper: ‘One empirical finding which is not directly anticipated in either the economics of organisation or the inter-organisational relationships literatures was the critical importance of organisational capacity. Strategic purchasing is technically demanding and requires a number of capacities of the purchaser.’^1^ The realist review from which our paper was derived included the issue of organisational capacity as a key aspect of strategic purchasing. Limitations of space prevented us from discussing it in the paper, but this correspondence provides an ideal opportunity to do so, albeit briefly.

Much of the procurement literature concerns buyer-supplier interactions, with principal-agent, transaction cost and inter-organisational relationships theories centre-stage. This was covered in our recent paper.^2^ However, there is also a complementary part of the literature that concerns ‘organisational buying behaviour’ (OBB) – research into the structures, cultures, systems, processes, capabilities and behaviours (that together create organisational capacity) that affect whether the buy-side organisation can manage its interactions with suppliers effectively. In what follows, we look at three key aspects of the OBB literature and illustrate their relevance to strategic purchasing via an application to English National Health Service (NHS) commissioning.

## OrganisationalBuyingBehaviourand Strategic Purchasing

### Procurement Set-up

There is a long-running debate within the procurement literature regarding how procurement activity should be organised. Two key issues concern the degree of centralisation of procurement^3^ and a related, although not synonymous, issue of the appropriate level of purchasing aggregation.^4^ The former issue has been the focus of much policy in the NHS regarding medical supplies, with many re-organisations at both a regional and national level.

More pertinent to NHS commissioning, however, is the issue of aggregation. There has been continual change in the size/patient coverage of commissioning bodies since the introduction of the purchaser-provider split in 1990. Initially, there was a policy of general practitioner (GP) fundholding, with 57% of GPs involved at the time of its abolition in 1998.^5^ GP fund-holders were replaced in 1998 by 481 primary care groups (PCGs),^6^ in turn replaced by 303 primary care trusts (PCTs),^7^ which were reduced to 151,^8^ then replaced by 212 clinical commissioning groups (CCGs),^8^ which are now due to be dramatically reduced in number to map better onto the integrated care systems developed as part of the reversal of the Lansley reforms of 2012.^9^

Familiar themes common within the OBB literature^3^ have been cited by NHS personnel in favour of different levels of aggregation. On the one hand, concerns have been expressed about a too high a level of aggregation. For example, Julie Wood, chief executive of NHS Clinical Commissioners, commented recently: ‘Clinical commissioners want to stay connected to their constituent places and for their systems to make sense for the populations they serve, which means it’s likely that the future number of CCGs to be more than the current 44 sustainability and transformation partnerships.’^9^

On the other hand, there has also been talk of the benefits of scale, in particular consistency, efficiency and buying power. For example, with respect to the merger of eight CCGs in London, it was claimed that the newer bigger entity would end ‘any suggestion of a postcode lottery by making services equitable across the eight boroughs’ and that financial savings would ‘come from reducing duplication.’^10^ The creation of PCTs to replace the larger number of PCGs was accompanied by the language of power, as PCTs were expected to negotiate effectively with hospital trusts.^11^

The NHS is, of course, not the only organisation to have struggled to find the right balance between levels of centralisation and aggregation, with swings between opposites commonplace.^3^ Its high political profile, however, does seem to make it hard for politicians to heed the commonly-heard refrain of ‘no more top-down re-organisations.’

### Procurement Capability and Information

While the issue of procurement set-up is important – structures matter – it has been hard to judge whether any particular structural arrangements for commissioning over the past three decades were optimal (or indeed whether the purchaser-provider split was a good idea), as no NHS commissioning organisation has been given sufficient time to be properly established. This lack of time is one important reason why NHS commissioning organisations have fallen short of reaching the required level and quality of capabilities and information suggested by the OBB literature.

In terms of capabilities, effective procurement requires effective leadership within the procurement function^12^; high-quality procurement personnel, with different skill sets^13^ and an appropriate level of rotation^14^; and, developed team-working skills, both on the part of those in the procurement function and those outside it who provide input into the procurement process.^15^

In terms of information, there is a need for an effective buying organisation to develop accurate and usable information about the profile of the organisation’s demand. This is obvious, of course, but difficult for reasons of uncertainty, complexity and the vagaries of IT systems and their usage.^16,17^ There is also a need for accurate information on the often myriad supply markets a buying organisation interacts with. This is not just regarding the availability of suppliers, but also their capability^18^ and the level of bargaining power the buying organisation possesses relative to suppliers.^19^

The failure of NHS commissioners in this respect was outlined by the House of Commons Health Committee in 2010. A key conclusion of the report, which reflected on the performance of PCGs and PCTs between 1998 and 2010, was squarely about capabilities and management information: ‘Weaknesses are due in large part to PCTs’ lack of skills, notably poor analysis of data, lack of clinical knowledge and the poor quality of much PCT management. The situation has been made worse by the constant re-organisations and high turnover of staff.’^20^ This poor OBB was said to have consequences: ‘[R]ecent NAO value for money reports … highlighted weaknesses at PCT level in all three stages of the commissioning cycle: strategic planning, procuring services and monitoring and evaluation.’^20^

### Procurement Politics and Governance

A third aspect of the OBB literature concerns cross-functional co-operation (or lack of it) and procurement governance. It has, of course, been widely argued that organisations are affected by power and politics.^21^ It has also been noted that the procurement process within organisations is not exempt from this.^4^ Commercial and non-commercial managers within procuring organisations can disagree over the priorities and specifics of a procurement exercise, leading to demand-side problems such as product or service over-specification and poor service scoping.^22^ Such disagreement can also lead to problems with ‘procurement governance’ in the negotiation and contract management stages of the process – that is, either confusion or disagreement of over the allocation of roles and responsibilities.^23^ Various responses to this, especially stakeholder engagement, have been suggested to address the conflicts and problems.^12^

The dynamics of power and politics have affected NHS commissioning. For example, Vize^7^ commented on the operation of PCTs: ‘In theory, clinicians were well represented on the commissioning side … But too often there was a distant, or even antagonistic, relationship between local GPs and PCT management. This failure to bring an authentic clinical voice to PCT strategies made it more difficult for commissioners to engage clinical staff in the trusts.’ A major hope for the replacement CCGs was that these relationships would be better. In that spirit, David Smith, a chief officer in a CCG, provided advice to CCGs regarding stakeholder engagement: ‘If you really want to be effective locally you must understand how your local authority works and you must put time and energy into cultivating these relationships … CCGs will also need to acquaint themselves with the local arrangements for health scrutiny …[G]et to know the chair of their health scrutiny committee, understand their concerns and agree how they are going to work with them.’^24^

### Conclusions: Taking OBB Seriously at an Organisational and Policy-Level

Much of the OBB literature is focused at the organisation-level and this work resonates with the experience of NHS commissioning organisations over the past 20 years. However, as the brief discussion above shows, there is also a need for the lessons of the OBB literature to be understood and applied at a policy-level. There were always reasons related to the structure of the secondary care sector for believing that the NHS purchaser-provider split would struggle to provide the improvements in efficiency and effectiveness expected in government and think-tanks. However, there is little doubt that the limited achievements of the policy have been exacerbated by the fact that at no stage over the past three decades has any UK government taken the lessons of OBB seriously. As Hanson et al^1^ were correct to point out, such lessons are as important as the guidance provided by the economics of organisation and inter-organisational relationships literatures, and they need to be given equal weight in understanding what’s needed to develop successful strategic purchasing in healthcare.

## Ethical issues

Not applicable.

## Competing interests

Authors declare that they have no competing interests.

## Authors’ contributions

All authors conceived the idea for the original funded realist review on which this paper is based, with JS being the lead investigator. CL wrote the first draft of this manuscript. All authors provided input into ongoing iterations of the manuscript and approved the final version.

## Authors’ affiliations

^1^Birmingham Business School, University of Birmingham, Birmingham, UK. ^2^Health Services Management Centre, University of Birmingham, Birmingham, UK.
